# Health care for people with tuberculosis/HIV co-infection from the multidisciplinary team’s perspective

**DOI:** 10.1590/0034-7167-2022-0733

**Published:** 2023-10-09

**Authors:** Eduarda Aguiar da Silva, Paula Hino, Hugo Fernandes, Maria Rita Bertolozi, Aline Aparecida Monroe, Lucimara Fabiana Fornari

**Affiliations:** I Universidade Federal de São Paulo. São Paulo, São Paulo, Brazil; II Universidade de São Paulo. São Paulo, São Paulo, Brazil

**Keywords:** Tuberculosis, HIV Infections, Coinfection, Public Health, Patient Care Team., Tuberculosis, Infecciones por VIH, Coinfección, Salud Pública, Grupo de Atención al Paciente., Tuberculose, Infecções por HIV, Coinfecção, Saúde Pública, Equipe de Assistência ao Paciente.

## Abstract

**Objective:**

to know the multidisciplinary team’s perspective about the health care of people with tuberculosis and human immunodeficiency virus co-infection in relation to treatment.

**Methods:**

this is a descriptive-exploratory study, with a qualitative approach, carried out in a health care service in São Paulo, from May to June 2019. Semi-structured interviews were conducted with nine professionals from the multidisciplinary team. Data were processed through discourse analysis with the support of webQDA.

**Results:**

Two empirical categories emerged: Health care interfaces for people with tuberculosis and human immunodeficiency virus co-infection; Barriers and facilitators for health care for people with co-infection.

**Final considerations:**

the health-disease process in co-infection is mediated by conditions that positively or negatively interfere with treatment compliance. People’s health care goes beyond exclusively clinical assistance and requires the recognition of needs in a broad perspective.

## RESUMO

**Objetivo:**

conhecer as percepções da equipe multiprofissional sobre cuidado em saúde de pessoas com coinfecção tuberculose e vírus da imunodeficiência humana em relação ao tratamento.

**Métodos:**

estudo descritivo-exploratório, com abordagem qualitativa, realizado em serviço de assistência à saúde de São Paulo de maio a junho de 2019. Foram realizadas entrevistas com roteiro semiestruturado com nove profissionais da equipe multiprofissional. Os dados foram processados por meio da análise de discurso com apoio do *software* webQDA.

**Resultados:**

duas categorias empíricas emergiram: *Interfaces do cuidado em saúde à pessoa com coinfecção tuberculose e vírus da imunodeficiência humana; Barreiras e facilitadores para o cuidado em saúde à pessoa com coinfecção*.

**Considerações finais:**

o processo saúde-doença na coinfecção é mediado por condicionantes que interferem de forma positiva ou negativa na adesão ao tratamento. O cuidado em saúde das pessoas ultrapassa a assistência exclusivamente clínica e requer o reconhecimento de necessidades em uma perspectiva ampla.

## RESUMEN

**Objetivo:**

conozca las percepciones del equipo multiprofesional sobre el cuidado de la salud de las personas con tuberculosis de coinfección y el virus de la inmunodeficiencia humana en relación con el tratamiento.

**Métodos:**

estudio descriptivo-exploratorio, con un enfoque cualitativo, realizado en servicios de atención médica de São Paulo de mayo a junio de 2019. Se realizaron entrevistas con guiones semiestructurados con nueve profesionales del equipo multiprofesional. Los datos se procesaron a través del análisis del discurso con el soporte del software webQDA.

**Resultados:**

surgieron dos categorías empíricas: interfaces de atención médica para persona con tuberculosis de coinfección y virus de inmunodeficiencia humana; Barreras y facilitadores para la atención médica para personas con coinfección.

**Consideraciones finales:**

el proceso de enfermedad de salud en la coinfección está mediado por condiciones que interfieren positiva o negativamente en la adherencia al tratamiento. La atención médica de las personas va más allá de la asistencia clínica exclusiva y requiere el reconocimiento de las necesidades desde una perspectiva amplia.

## INTRODUCTION

Tuberculosis (TB) remains a public health problem, representing the main isolated cause of infectious death in the world until the advent of the COVID-19 pandemic. Globally, it is estimated that, in 2020, there were 9.90 million TB infections, of which 792,000 (8%) were cases associated with the human immunodeficiency virus (HIV). Worldwide, there were approximately 1.3 million deaths from TB, 214,000 of which among people living with HIV (PLHIV), in which TB is the main cause of death^(1)^.

Brazil appears in two of the three lists of 30 priority countries for the development of actions to contain TB in the period between 2021 and 2025: those with the highest incidence of the disease and those with the highest numbers of cases among PLHIV^(1)^.

However, it should be noted that since the World Health Organization (WHO) declared TB a global public health emergency in 1993, Brazil has been developing several strategies to control the disease. In 2017, the country launched the Brazilian National Plan to End Tuberculosis, which represents a guiding document for actions to combat the disease and whose goal is to reduce the incidence rate to less than ten cases per 100,000 inhabitants and to reduce the mortality rate to less than one death per 100,000 inhabitants by 2035^(2)^. Furthermore, the agreed strategies related to TB control in PLHIV aim to intensify collaborative TB-HIV activities^(3)^.

Regarding TB/HIV co-infection, PLHIV are 28 times more likely to develop active TB compared to the general population^(4)^. In 2020, as a result of the COVID-19 pandemic, there was a 10.9% drop in the notification of new TB cases compared to the same period of the previous year in Brazil, with 68,700 new cases of TB being registered, that of these, 8.4% were PLHIV^(3)^.

People with TB/HIV co-infection face numerous adversities related to the health-disease process, which can result in failures in treatment compliance, such as quantity of medication, occurrence of side effects, lack of motivation, feeling of anxiety, among others^(5)^. Treatment non-compliance and inappropriate medication use can trigger serious consequences, predictors of unsatisfactory treatment results and contribute to the development of drug-resistant TB (DR-TB): one of the obstacles to disease control^(6)^.

Treatment compliance is complex, as it is not restricted to the mere intake of medication, but involves aspects related to living and working conditions, inherent to the health-disease process itself. In this sense, this study used the concept of compliance based on three interdependent plans: 1) what refers to the knowledge about the health-disease process presented by a person who has the disease; 2) what is the social place occupied by a sick person; and 3) what refers to the health production system, more specifically in relation to organization of health in order to allow broad access to care. It goes beyond, therefore, the perspective of treatment abandonment, limited to a restrictively individual attitude or behavior^(7)^.

This refers to the need to consider the health-disease process as a social phenomenon, contemplating the needs for coping with the disease^(8)^. For this to happen, health professionals need to establish a bond with people with TB and their family, in order to identify and understand situations that may compromise treatment compliance, such as precarious living conditions, indiscriminate use of alcohol and drugs, and weaknesses in the support network^(9)^.

Several studies have investigated treatment compliance in TB/HIV co-infection^(10-15)^, whose results motivated this study, which had as its central question: what are multidisciplinary team’s perceptions about the care provided to people with HIV who undergo TB treatment?

## OBJECTIVE

To know the multidisciplinary team’s perceptions about health care for people with TB/HIV co-infection in relation to treatment.

## METHODS

### Ethical aspects

In the case of research involving human beings and in compliance with ethical and legal precepts, the study complied with the norms of Resolution 466/2012, and was approved by the Research Ethics Committees of the *Universidade Federal de São Paulo* and the aforementioned health service where data collection took place. Health professionals who agreed to participate in the research signed the Informed Consent Form.

### Study design

This is a descriptive-exploratory study, with a qualitative approach, since the main purpose was to explore the multidisciplinary team’s perceptions that works in health care of people with TB/HIV co-infection. The study followed the Consolidated criteria for REporting Qualitative research (COREQ) recommendations^(16)^.

### Study setting

The study setting was a state reference and training center on HIV/AIDS located in the city of São Paulo, which provides medical-hospital, outpatient and home care to people with sexually transmitted infections and HIV/AIDS, in addition to carrying out prevention activities, epidemiological surveillance, management and research.

### Data source

Nine health professionals from the multidisciplinary team of a state reference and training center on HIV/AIDS, located in the city of São Paulo, participated in the study. Professionals who had worked for at least three months in the referred health service and worked in the care of people with TB/HIV co-infection were included, given the possibility of having more experience in the development of care practices for this group. Exclusion criteria for participation in the study were not defined. The study sample was constituted by convenience and defined by saturation in qualitative research, i.e., as there was recurrence and complementarity of information^(17)^.

### Data collection

Data came from an interview conducted using a semi-structured script prepared by the authors of this study. The first part of the script presented information related to participant sociodemographic data and the second involved questions related to the theme of this study, guided by the main question: what are your perceptions about the care offered to PLHIV who undergo treatment for TB?

Data collection was carried out by a researcher who received training to conduct the interviews and took place from May to June 2019. Professionals who provided care to PLHIV undergoing TB treatment were invited to participate in the study. They were personally approached during working hours and interviews were subsequently scheduled according to participants’ availability.

The interviews were carried out individually, in a reserved place on the health service premises, in order to guarantee ease of access, privacy, comfort and quality of information. The interviews were recorded using a digital audio recorder to seek greater authenticity in understanding the statements, with an average duration of 25 minutes. Subsequently, the audios were transcribed in full by the interviewers themselves, and participants’ names were replaced by the letter “E” followed by an Arabic number to guarantee anonymity.

### Data analysis

The testimonies were submitted to interpretation using the discourse analysis method, which allows the construction of empirical categories. This method of analysis seeks to understand the meanings that people express through their speech, constituted by ideology, history, language^(18)^ and representations about everyday life. As TB is an infection that is known to have a social nature, statement analysis was carried out in the light of the Theory of Social Determination of the Health-Disease Process.

The Theory of Social Determination of the Health-Disease Process proposes a dialectical interpretation of social life by considering that health is a complex process that involves a movement between determination and relative autonomy^(19)^.

Discourse analysis was carried out with the support of webQDA qualitative analysis software^(20)^, in order to optimize qualitative data treatment and management. The interviews transcribed in full were inserted into the internal sources system. Through the Coding system, the interviews were classified according to profession, gender, marital status, age group, education, time working in the service and time working with people co-infected with participants’ TB/HIV. The empirical categories were constructed using Tree Codes, which resulted in the identification of two empirical categories*: Health care interfaces for people with tuberculosis/HIV co-infection; Barriers and facilitators for health care for people with tuberculosis/HIV co-infection*.

## RESULTS

Among the totality (n=9) of study participants, three were nurses, two, nursing technicians, one, a doctor, one, a psychologist, one, a social worker, and one, a pharmacist. Most participants were female (n=7), aged between 40 and 49 years old (n=5), followed by those between 50 and 59 years old (n=4). Regarding working time dedicated to caring for people with TB/HIV co-infection, an average experience of 18.3 years was observed for nurses, five, for nursing technicians, 10, for the doctor, four, for the psychologist, 20, for the social worker, and 29, for the pharmacist.

**Figure 1:**
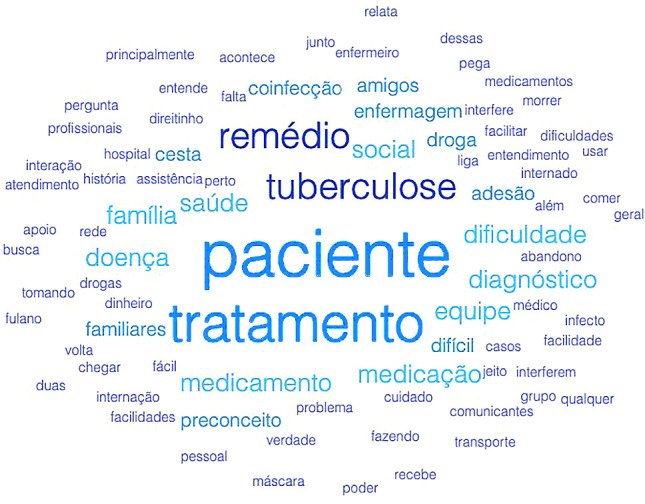
Cloud of the 100 most frequent words in the interviews

In the interviews, as can be seen in the word cloud created using the webQDA software (Figure 1), the most frequent words were patient (n=203), treatment (n=164), TB (n=101), medication (n=95), health (n=65), medication (n=64), team (n=59), family (n=52), diagnosis (n=47), compliance (n=43) and coinfection (n=37).

Discourse analysis enabled identifying meanings and senses about health care of people with TB/HIV co-infection from the perspective of treatment compliance, and enabled identifying two empirical categories*: Health care interfaces for people with tuberculosis/HIV co-infection*; and *Barriers and facilitators for health care for people with tuberculosis/HIV co-infection* (Chart 1).

**Chart 1:** Categories and themes identified through empirical data

Empirical categories	Themes addressed
Health care interfaces for people with tuberculosis/HIV co-infection	- Medication treatment - Care strategies - User attention network
Barriers and facilitators for health care for people with tuberculosis/HIV co-infection	- Barriers: social vulnerability, chemical dependency and stigma and prejudice - Facilitators: family support, access to information and prognosis

The empirical categories and the respective discourses are presented below:

### Health care interfaces for people with tuberculosis/HIV co-infection

According to the multidisciplinary team, a portion of users have difficulty complying with treatment due to side effects and drug interactions.

*Yes, the drugs interact. These are drugs that potentially are not drugs that are easy to handle.* (E5)

*Medications cause many side effects, even more associated with antiretrovirals and other diseases that can have neurotoxoplasmosis, neurocryptococcosis, and ends up interfering in this whole context.* (E7)

The pathological process of the two infections was also pointed out, since it weakens the body and accentuates some signs and symptoms of TB, such as loss of weight and appetite, stomach pain and weakness. In these cases, the importance of social incentives for carrying out treatment was emphasized:

*There is drug interaction and they feel very debilitated with TB and HIV drugs. That’s where you need to go with the basic food basket to see if they eat a little bit, get a little stronger to withstand treatment.* (E1)

The dynamics of medication supervision varied according to each user’s particularities. In cases where there was difficulty in complying with treatment, the multidisciplinary team reported performing directly observed treatment (DOT) at the reference center itself or at the primary care units, scheduling more frequent returns and telemonitoring to observe medication intake. In view of this, continuous follow-up to monitor clinical condition was highlighted, usually carried out through medical consultations and laboratory tests.

Additionally, the multidisciplinary team highlighted as health care strategies the provision of basic food baskets and transportation assistance, especially for users with unfavorable socioeconomic conditions. The importance of embracing users and their families to provide guidance on treatment, disease, transmission, stigma and prejudice was also mentioned. Moreover, participants mentioned the need to establish a bond with users to build a unique therapeutic plan, in order to encourage co-responsibility in the therapeutic process.

*A basic food basket and driving aid are offered. For some patients, lunch is offered here, so they come close to lunchtime, take the medication and then stay for lunch.* (E6)

*I was improving her relationship with the institution, the relationship with the doctor was great, with me, with lunch, with coming regularly, she was improving the bond and this meant that she had a regularity to be treated in the first two years.* (E8)

The Health Care Network was highlighted with emphasis in the context of treatment and monitoring of users with TB/HIV co-infection. In this regard, speeches demonstrated that each professional plays a specific role in the therapeutic process. Furthermore, the importance of clinical discussions involving the multidisciplinary team was highlighted so that professionals make decisions in line with the possibilities of each case. It was also noted that there was cooperation between the referred health service and primary and hospital care units, which enabled more effective interventions in more complex cases.

*The doctor does not take care of a patient alone. They have to communicate, when they do, everyone knows, before starting treatment. Nursing already maps where he lives to see the nearby units that can support treatment, which ones have the medicine, sometimes they have to send the medicine from here to there.* (E8)

*He was admitted to the hospital in Araraquara for treatment of people with multidrug-resistant tuberculosis and we made all the contact, we worked in a network, we went there to discuss the case.* (E4)

### Barriers and facilitators for health care for people with tuberculosis/HIV co-infection

Among the barriers to health care, the multidisciplinary team cited social vulnerability, resulting from the precariousness of housing, food and transportation, in addition to the fragility of income for maintaining subsistence.

*I think it’s more the social part, as we said, it’s homeless, the person is poor and unemployed, there’s no way to take a bus, a subway to get here, sometimes they live very far away.* (E3)

*It’s no use having a nice place like this, with medicine and a laboratory, if the user says, “Doctor, I have nowhere to sleep or eat, you told me to come back next week, but I don’t have the money to come”. It has an important social demand and TB/HIV co-infection is generally a disease of poorer people.* (E5)

Another barrier in health care refers to chemical dependency, which interferes with the regularity of the bond with the health service.

*Patients using drugs. Illicit drugs do make it difficult. They often prefer to spend nights on the street, three days on the street. Doesn’t come home or doesn’t go to the BHU, so it does interfere.* (E1)

*The biggest difficulty is because there is the issue of drug craving, drug use and then it relapses, it goes back to the street and it is lost again. So this is a very complex demand.* (E4)

In addition to these issues, there is evidence of prejudice within the family and social spheres and the stigma experienced by people with TB/HIV co-infection. The reports demonstrated the existence of barriers to acceptance of the clinical condition even by the users themselves, since they constantly suffered situations of prejudice:

*He doesn’t comply with because he was rejected by the family, the family built a doghouse for him, he can’t eat, there’s no way to treat himself and it gets worse every day because he can’t comply with.* (E9)

*Prejudice comes from the house, in the family itself. Some parents sometimes do not accept it, especially if they are a transvestite or an HIV carrier.* (E1)

On the other hand, participants’ speeches denoted the positive impact of situations involving family support in treatment compliance.

*When the family is present, of course, it makes it much easier, because family support makes all the difference.* (E7)

*The question, for example, of you having regular hours, of having a place to store the medication, of having someone to remind you to take this medication […].* (E4)

Another point that was present in the interviews was the fact that users with greater access to information found it easier to accept the therapeutic plan and, consequently, had a favorable prognosis in TB/HIV co-infection.

*Just go to any health center, a BHU, and say, “I want to do a test, but I don’t want anyone to know”, it’s very easy. It seems that the number of people with HIV has increased, it’s not because they are uninformed, it’s because the media is showing how easy it is to prevent it every day* […]. (E2)

*When they look at the number of pills, it is not a small problem, but it will end, so the idea that it will end, that there is a discharge, that there is a cure, it relieves everything, so there is no stigma.* (E8)

## DISCUSSION

Discourse analysis clarified issues that influenced the health-disease process of people who experience TB/HIV co-infection. From the multidisciplinary team’s perspective, socioeconomic fragility, precarious living conditions and low education have significant relevance in this process, as they reflect on the difficulty of accessing care and health services as preponderant points for following the therapeutic process.

On the other hand, a study carried out in Nigeria demonstrated the absence of this association^(15)^, which may instigate other studies on the subject in this perspective, in addition to highlighting issues that may not have been addressed in this study and that indicated the relevance of cultural issues that favored treatment compliance.

A study carried out in Africa revealed that, among the difficulties related to TB treatment non-compliance among PLHIV, personal beliefs, fear of side effects, lack of knowledge about treatment and its importance, in addition to use of alternative medicine practices, stood out. It is also important to understand other constraints, such as geographical distance from health units, lack of assistance programs and psychosocial and family aspects^(21)^.

In addition to issues related to the structural and particular dimensions of society, participants mentioned the unique dimension of each user. From this perspective, the multidisciplinary team considered dependence on alcohol and other drugs as a barrier to the therapeutic process, a result corroborated by a study carried out in Fortaleza based on the analysis of data obtained from the Information System for Notifiable Diseases (SINAN - *Sistema de Informação de Agravos de Notificação*)^(11)^.

Social and family support, health professionals embracing users and their families, health education and social incentives were cited as positive aspects that can promote treatment compliance. It is understood that, for treatment compliance to occur, professionals need to understand that the health-disease process transcends multifactoriality, being understood in the light of constraints related to the structure of society, social reproduction, in addition to understanding the uniqueness of each person and their life process in the therapeutic plan.

Research on treatment compliance from the perspective of people who experienced TB/HIV co-infection found that the bond with the health team, humanized embracement, provision of a basic food basket, the possibility of withdrawing medications from the health service and multidisciplinary follow-up favored treatment compliance. Thus, the importance of implementing actions that guarantee comprehensive and humanized care is highlighted, considering the health needs of PLHIV undergoing TB treatment^(10)^.

A qualitative study carried out with Primary Health Care professionals in the city of São Paulo identified social incentives, DOT and bond between user and health team as positive points. However, the authors highlighted that, despite the incentives favoring treatment compliance, public measures are needed to overcome social inequalities and consequent changes in living conditions, especially for the population living in a situation of social vulnerability^(22)^.

It is considered that the health team must be able to embrace users, taking into account each one’s particularity, the knowledge, beliefs, difficulties faced, aiming to seek, together with users, ways to overcome the barriers to follow-up treatment^(9)^. In this regard, health education actions make it possible to expand knowledge and allow strengthening the bond between user and health professional. It is noteworthy that the moment of disclosure of diagnosis is crucial for users’ lives and it is when the embracement and bond between health professional and user begins, aiming at a favorable impact on treatment compliance^(23)^.

A study carried out with health professionals in the city of Rio de Janeiro analyzed the care offered to people with TB, showing that the support and bond with the professionals who provided care had a positive influence on treatment compliance^(24)^.

It is essential that, in order to achieve the expected results, health services are structured in order to facilitate diagnosis and access to medications^(6)^. The combined treatment of the two infections proved to be a complex issue for the therapeutic process, since the amount of pills to be ingested daily can impact continuity of treatment^(14)^. Moreover, adverse effects were another negative point evidenced in the speeches and corroborated by a study carried out with people undergoing treatment for multidrug-resistant TB in the city of São Paulo^(25)^.

The difficulty in accessing health care and the consequent delay in starting treatment may be related to the mortality of people with TB/HIV co-infection. Therefore, the importance of optimizing treatment outcomes in TB and HIV programs is highlighted, particularly by improving early HIV diagnosis, early initiation of antiretroviral therapy (ART) and TB treatment compliance^(12)^.

It was learned from participants that people with TB/HIV co-infection have difficulties in coping with their health condition, mainly due to the stigma and prejudice experienced in the social and family environment, with negative repercussions on treatment compliance. Still in relation to the study, the impact of health education stands out, as a provider of treatment compliance, since it allowed users to understand their health condition, which made them active in the therapeutic process. In fact, studies have demonstrated the effectiveness of counseling in continuing treatment^(13,16)^.

A study carried out in Africa on ART acceptance by people undergoing TB treatment showed that the socioeconomic and individual barriers most frequently described were stigma, low income and younger age group. Barriers related to the health service were scarcity of human resources, lack of infrastructure and treatment guidelines. Clinical barriers included anti-TB drug intolerance, fear of drug toxicity, and antiretroviral contraindications^(13)^.

It is noteworthy that participants did not mention problems related to the lack of human resources, infrastructure and drug treatment. Barriers were mainly centered on user demands. Therefore, it is important to suggest carrying out a study from service users’ perspective to verify whether barriers are similar to what was mentioned by the health team.

A qualitative study carried out with doctors and nurses who worked in an African community with a high number of cases of TB and HIV pointed to the need to implement measures aimed at the effective TB/HIV co-infection treatment, as improving co-infection management through effective policies, training of health staff, adequate infrastructure and establishing partnerships between stakeholders, and encouraging research^(25)^.

Regarding the findings of this study, participants pointed out the need to create strategies that mitigate the barriers experienced by users, in order to guarantee treatment compliance, such as offering incentives. Furthermore, the importance of considering health professionals’ work is highlighted, in order to produce better treatment results and that TB and HIV/AIDS control programs act in an integrated manner.

Therefore, in the light of the Social Determination of the Health-Disease Process, it is emphasized that treatment depends on several conditions, since access to health actions and services is not enough to overcome the problem associated with user compliance. Thus, it is understood that the health-disease process is a social phenomenon and requires meeting users’ and families’ health needs, in order to offer responsible and qualified care^(8)^.

### Study limitations

It is pointed out as a limitation of this study the performance of interviews with professionals from only one health service. However, although it is not possible to generalize the findings, it is believed that the results can contribute to a better understanding of the health care offered to people with TB/HIV co-infection from health professionals’ perspective.

### Contributions to health

It is hoped that the findings of this study will allow a theoretical deepening of the range of aspects that influence care practices aimed at TB/HIV co-infection treatment and, thus, bring subsidies to rethink the health practices developed for this specific group.

## FINAL CONSIDERATIONS

According to participants’ interpretive perspective, the health-disease process in TB/HIV co-infection resulted from several conditions that interfered positively or negatively in the effectiveness of treatment. Therefore, social and political efforts to reduce health inequalities, as well as health actions that overcome aspects related to programmatic vulnerability, contribute to treatment compliance. It is also believed that the participation of users in the therapeutic process is crucial to overcome the barriers inherent in the experience of co-infection.

## Data Availability

https://doi.org/10.48331/scielodata.DD0JNX
